# Supplementary material for: Modelling Genetic Benefits and Financial Costs of Integrating Biobanking into the Captive Management of Koalas
Source: Animals (Basel). 2022 Apr 12;12(8):990. doi: 10.3390/ani12080990 (PMC9028793; doi:10.3390/ani12080990)
Supplement: Supplementary file 1 [file animals-12-00990-s001.zip › animals-1620303-supplementary.pdf]

animals

MDPI

Article

Modelling Genetic Benefits and Financial Costs of Integrating Biobanking into the Captive Management of Koalas

Lachlan G. Howell [1,2,3,*], Stephen D. Johnston [4], Justine K. O'Brien [5], Richard Frankham [6], John C. Rodger [2,3], Shelby A. Ryan [2,3], Chad T. Beranek [2,3], John Clulow [2,3], Donald S. Hudson [7] and Ryan R. Witt [2,3,*]

1 Centre for Integrative Ecology, School of Life and Environmental Sciences, Deakin University Geelong, Melbourne Burwood Campus, 221 Burwood Highway, Burwood, VIC 3125, Australia
2 School of Environmental and Life Sciences, Biology Building, University of Newcastle, University Drive, Callaghan, NSW 2308, Australia; john.rodger@newcastle.edu.au (J.C.R.); shelby.ryan@uon.edu.au (S.A.R.); chad.beranek@newcastle.edu.au (C.T.B.); john.clulow@newcastle.edu.au (J.C.)
3 FAUNA Research Alliance, P.O. Box 5092, Kahibah, NSW 2290, Australia
4 School of Agriculture and Food Sciences, The University of Queensland, Gatton, QLD 4343, Australia; s.johnston1@uq.edu.au
5 Taronga Institute of Science and Learning, Taronga Conservation Society, Bradleys Head Rd., Mosman, NSW 2088, Australia; jobrien@zoo.nsw.gov.au
6 School of Natural Sciences, Macquarie University, Sydney, NSW 2109, Australia; richard.frankham@mq.edu.au
7 Port Stephens Koala & Wildlife Preservation Society LTD., t/a Port Stephens Koala Hospital, One Mile, NSW 2316, Australia; don.hudson@pskh.com.au
* Correspondence: l.howell@deakin.edu.au (L.G.H.); ryan.witt@newcastle.edu.au (R.R.W.)

Table S1. Captive holding/management costs for koalas used in cost modelling presented in this study. The table includes each itemized cost including a description. The costs are presented per individual animal. Itemized costs are presented as either fixed or variable. The frequency at which individual cost components recur in long-term cost modelling is also shown (based on the effective life of depreciating assets as per the Australian Taxation Office: https://www.ato.gov.au/law/view/document?docid=TXR/TR20195/NAT/ATO/00003 access date: 29 March 2022). All dollar amounts are shown in Australian currency (A$).

Costs for conventional koala captive management used for modelling	Description	Costs (per individual animal)	Fixed or variable	Recurrent costs frequency (year intervals)*
Annual captive holding cost per individual animal	Annual average holding cost per individual koala (including veterinary, husbandry, eucalypt plantation management, genetic management and staff).<br><br>Value based on expert practitioner knowledge from Steve Johnston and the former Dreamworld Life Science Manager.<br><br>Note: does not include infrastructure and facilities costs.	$12,000	Variable	1 (every year)
On-site infrastructure/facilities costs	Koala enclosure. On-site infrastructure. 4-8 koalas. Between $60-80K. Year 1.	$11,667	Variable	20

**Total Set-up**	Per individual cost is based on the median number of 6 koalas applied to the median cost of $70k. n.d	**$23,667**	n.d	n.d

Table S2. Costs specific to the integration of biobanking and assisted reproductive technologies used in the cost modelling presented in this study. The table includes each itemized cost including a description. Itemized costs are presented as either fixed or variable. The Year 1 costs are shown as well as the frequency at which these same individual cost components recur in long-term cost modelling (based on the effective life of depreciating assets as per the Australian Taxation Office: https://www.ato.gov.au/law/view/document?docid=TXR/TR20195/NAT/ATO/00003; access date: 29 March 2022). All dollar amounts are shown in Australian currency (A$).

Component of biobanking system for backcrossing	Description	Year 1 Costs	Fixed or variable	Recurrent costs frequency (year intervals)
Anesthetic machine – isoflurotec	Required to sedate koalas during semen collection or assisted reproductive procedures.	$10,000	Fixed	10
Phase contrast microscope	Used to assess and manage semen samples.	$15,000	Fixed	10
Electroejaculation equipment and probes	Required to collect sperm from koalas in the field and in the hospital.	$10,000	Fixed	6
Laparoscope	Required for ultrasound guided oocyte aspiration (oocyte collection) and embryo transfer.	$20,000	Fixed	10
Benchtop incubator (GERI) and culture media	Required for oocyte and embryo culture.	$50,000	Fixed	7
Stereoscope and stage warmer	Required for intracytoplasmic sperm injection.	$12,000	Fixed	10
Anti-vibration table, micromanipulator controllers and consumables	Required for intracytoplasmic sperm injection.	$78,000	Fixed	10
Air filtration unit (Coda) plus filters	Required for intracytoplasmic sperm injection and embryo culture.	$5,000	Fixed	10
Portable incubator for oocytes and embryos	Required for transport of oocytes and embryos.	$5,000	Fixed	7
Fridge/freezer for media, extenders	Required for frozen storage.	$800	Fixed	10
Consumables	e.g., Liquid Nitrogen, hormones, french straws.	$54,000	Fixed	n.d
Biobanking equipment & sample quality assurance consumables	e.g., sterile straws/vials, straw sealant balls, diluent reagents, sperm chromatin stability assays, viability stains/activation reagents, semen and cryodiluent filters, and liquid nitrogen.	$15,610	Fixed	n.d
Dewars and freezers	Required for the frozen and chilled storage of sperm.	$50,000	Fixed	10

Additional Founder collection	Required for the supplementary collection of founder sperm.	$20,000	Fixed	n.d
Total Set-up	n.d	$345,410	Fixed	n.d
Backcross costs (labor) total / yr	Reproductive technician (1.0x full-time equivalent) (to perform all backcross procedures using either backcross tool; intracytoplasmic sperm injection or artificial insemination; to perform all sperm cryopreservation and thawing): $94,651.<br><br>Scientific Officer (0.5 full-time equivalent) (to oversee all reproductive technology procedures , ovary culture, oocyte maturation, intracytoplasmic sperm injection, and sperm cryopreservation): $70,000.	$164,651	Fixed	1 (every year)

We adjusted previous iterations of the genetic backcrossing model presented for amphibians [1,2] and other marsupial species [3] to account for the unique challenges and characteristics of *P. cinereus* captive management, including reproductive complexity, life history traits, increased holding costs and requirements, generation length, backcross frequency, and effective population sizes (N_e).

Table S3. Genetic modelling assumptions and parameters for captive colonies of koalas. Includes general assumptions for genetic modelling already presented in previous iterations [1–3], as well as species-specific parameters used to adapt this modelling approach to suit the koala.

**Genetic modelling assumptions and parameters for captive colonies of koalas (*Phascolarctos cinereus*). Includes general assumptions for genetic modelling already presented in previous iterations [1–3], as well as species-specific parameters used to adapt this modelling approach to suit the koala.**
Model assumes *P. cinereus* founder males and females in the colony have $F_0 = 0$.
The captive colony would initially contain one live female per founder male (drawn at random from *P. cinereus* source populations). Under backcrossing scenarios, males produced each generation are redundant after founder sperm are banked from the F_0 generation.
Colony census numbers (*N*) are managed at each generation to achieve the target N_e (N_e assumed constant).
An average 1:1 sex ratio is assumed and achieved by colony management. An average of one female offspring per female contributed to subsequent generations (via management of the colony).
Random pairing of males and females occur each generation during back-crossing. Live colony pairings are uncontrolled (to be consistent with assumptions for published N_e values).
Each generation produced would entirely replace previous captive generation with no overlap.
Females in each non-backcrossed generation have the same variability in offspring numbers as do wild females.

At each backcrossing event one fertile female offspring is retained from each female in the colony. Male offspring are redundant and removed.<br><br>All biobanked founder males are unrelated ($F_0 = 0$).

Novel species-specific genetic modelling assumptions and parameters for captive colonies of *P. cinereus*
The frequency of *P. cinereus* females in the captive colony successfully breeding at a mean generation length of ~7 years has the same variability as wild females.<br><br>All *P. cinereus* females in the captive colonies are optimally sexually mature at 2 years of age, enabling artificial insemination and intracytoplasmic sperm injection to be performed on captive females at the appropriate mean generational interval of ~7 years. Male *P. cinereus* are not sexually mature until ~2 years of age necessitating their collection from the wild when mature or keeping in captivity until at least 2 years of age.<br><br>These values represent the *ex situ* age at optimal breeding for each species based on husbandry and published values [4] and a published assumed ~7-year generation length [5]. Here we assume that all individuals would be optimally sexually mature and breeding by this age.

Table S4. Costs for the integration of biobanking into the nodes presented in Figure 2. The table includes each itemized cost including a description. Itemized costs are presented as either fixed or variable. The Year 1 costs and on-going costs are shown. These costs are specific to the collection and storage of koala founder sperm and have been isolated from the detailed costs in Table S2.

Itemized costs for biobanking systems at each location across the mapped node network (Figure 2)	Description	Year 1 costs	On-going costs	Fixed or variable
Anesthetic machine – isoflurotec **	Required to sedate koalas during semen collection or assisted reproductive procedures.	$10,000	n.d	Fixed
Electroejaculation equipment and probes	Required to collect sperm from koalas in the field and in the hospital.	$10,000	n.d	Fixed
Fridge/freezer for media, extenders	Required for frozen storage.	$800	n.d	Fixed
Consumables	e.g., Liquid Nitrogen, hormones, french straws.	$54,000	n.d	Fixed
Biobanking equipment & sample quality assurance consumables	e.g., sterile straws/vials, straw sealant balls, diluent reagents, sperm chromatin stability assays, viability stains/activation reagents, semen and cryodiluent filters, and liquid nitrogen.	$15,610	n.d	Fixed
Dewars, dry shippers and freezers	Required for the frozen and chilled	$50,000	n.d	Fixed

Reproductive technician	storage of sperm and sample transport. (0.5x full-time equivalent) (to perform all sperm cryopreservation and thawing).	$47,326	$47,326/yr	Fixed
Total Set-up	n.d	$187,736	$47,326/yr	Fixed

Table S5. GPS locations of each organization revealed in the online search for the proposed integration of biobanking and assisted reproduction for the koala across the node network presented in Figure 2.

Node Organization	Latitude	Longitude	State of Australia
Friends of the Koala	-28.820818	153.302155	New South Wales
Port Macquarie Koala Hospital	-31.443247	152.919647	New South Wales
Port Stephens Koala Hospital	-32.76509	152.11442	New South Wales
Taronga Conservation Society Wildlife Hospital (Dubbo)	-32.273024	148.586592	New South Wales
Taronga Conservation Society Wildlife Hospital (Sydney)	-33.843177	151.239956	New South Wales
Moggill Koala Rehabilitation Centre	-27.576891	152.875033	Queensland
Currumbin Wildlife Hospital	-28.139845	153.48499	Queensland
Australia Zoo Wildlife Hospital	-26.834896	152.961458	Queensland
RSPCA Queensland Wildlife Hospital (Wacol)	-27.586065	152.922088	Queensland
RSPCA Queensland Wildlife Hospital (Eumundi)	-26.471882	152.963704	Queensland
Hidden Vale Wildlife Centre	-27.71638	152.463288	Queensland
Ipswich Koala Protection Society	-27.587849	152.595372	Queensland
Werribee Open Range Zoo Koala Hospital	-37.922345	144.667319	Victoria
Healesville Sanctuary Australian Wildlife Health Centre	-37.682651	145.535177	Victoria
Adelaide Koala & Wildlife Centre	-34.817141	138.558197	South Australia
Kangaroo Island Koala and Wildlife Rescue Centre	-35.786604	137.22901	South Australia

References

1. Howell, L.G.; Frankham, R.; Rodger, J.C.; Witt, R.R.; Clulow, S.; Upton, R.M.O.; Clulow, J. Integrating biobanking minimises inbreeding and produces significant cost benefits for a threatened frog captive breeding programme. *Conserv. Lett.* **2021**, *14*, e12776, doi:https://doi.org/10.1111/conl.12776.
2. Howell, L.G.; Mawson, P.R.; Frankham, R.; Rodger, J.C.; Upton, R.M.O.; Witt, R.R.; Calatayud, N.E.; Clulow, S.; Clulow, J. In-tegrating biobanking could produce significant cost benefits and minimise inbreeding for Australian amphibian captive breeding programs. *Reprod. Fertil. Dev.* **2021**, *33*, 573–587.
3. Howell, L.G.; Mawson, P.R.; Comizzoli, P.; Witt, R.R.; Frankham, R.; Clulow, S.; O'Brien, J.K.; Clulow, J.; Rodger, J.C. Modelling genetic benefits and financial costs of integrating biobanking into the conservation breeding of managed marsupials. *Conserv. Biol. In Review.*
4. McLean, N.; Handasyde, K.A. Sexual maturity, factors affecting the breeding season and breeding in consecutive

seasons in populations of overabundant Victorian koalas (*Phascolarctos cinereus*). *Aust. J. Zool.* **2007**, *54*, 385–392.
5. Johnson, R.N.; O'Meally, D.; Chen, Z.; Etherington, G.J.; Ho, S.Y.W.; Nash, W.J.; Grueber, C.E.; Cheng, Y.; Whittington, C.M.; Dennison, S. Adaptation and conservation insights from the koala genome. *Nat. Genet.* **2018**, *50*, 1102–1111.
